# Decline in Proliferation and Immature Neuron Markers in the Human Subependymal Zone during Aging: Relationship to EGF- and FGF-Related Transcripts

**DOI:** 10.3389/fnagi.2016.00274

**Published:** 2016-11-25

**Authors:** Christin Weissleder, Samantha J. Fung, Matthew W. Wong, Guy Barry, Kay L. Double, Glenda M. Halliday, Maree J. Webster, Cynthia Shannon Weickert

**Affiliations:** ^1^Schizophrenia Research Laboratory, Neuroscience Research AustraliaSydney, NSW, Australia; ^2^Schizophrenia Research InstituteSydney, NSW, Australia; ^3^School of Psychiatry, Faculty of Medicine, University of New South WalesSydney, NSW, Australia; ^4^School of Medical Sciences, Faculty of Medicine, University of New South WalesSydney, NSW, Australia; ^5^Garvan Institute of Medical Research, St. Vincent’s Clinical School and School of Biotechnology and Biomolecular Sciences, University of New South WalesSydney, NSW, Australia; ^6^Brain and Mind Research Institute, School of Medical Sciences, Sydney Medical School, University of SydneySydney, NSW, Australia; ^7^Neuroscience Research AustraliaSydney, NSW, Australia; ^8^Laboratory of Brain Research, The Stanley Medical Research InstituteKensington, MD, USA

**Keywords:** neurogenesis, subventricular zone, proliferation, aging, doublecortin, human, gliogenesis

## Abstract

Neuroblasts exist within the human subependymal zone (SEZ); however, it is debated to what extent neurogenesis changes during normal aging. It is also unknown how precursor proliferation may correlate with the generation of neuronal and glial cells or how expression of growth factors and receptors may change throughout the adult lifespan. We found evidence of dividing cells in the human SEZ (n D 50) in conjunction with a dramatic age-related decline (21-103 years) of mRNAs indicative of proliferating cells (Ki67) and immature neurons (doublecortin). Microglia mRNA (ionized calcium-binding adapter molecule 1) increased during aging, whereas transcript levels of stem/precursor cells (glial fibrillary acidic protein delta and achaete-scute homolog 1), astrocytes (vimentin and pan-glial fibrillary acidic protein), and oligodendrocytes (oligodendrocyte lineage transcription factor 2) remained stable. Epidermal growth factor receptor (EGFR) and fibroblast growth factor 2 (FGF2) mRNAs increased throughout adulthood, while transforming growth factor alpha (TGFα), EGF, Erb-B2 receptor tyrosine kinase 4 (ErbB4) and FGF receptor 1 (FGFR1) mRNAs were unchanged across adulthood. Cell proliferation mRNA positively correlated with FGFR1 transcripts. Immature neuron and oligodendrocyte marker expression positively correlated with TGFα and ErbB4 mRNAs, whilst astrocyte transcripts positively correlated with EGF, FGF2, and FGFR1 mRNAs. Microglia mRNA positively correlated with EGF and FGF2 expression. Our findings indicate that neurogenesis in the human SEZ continues well into adulthood, although proliferation and neuronal differentiation may decline across adulthood. We suggest that mRNA expression of EGF- and FGF-related family members do not become limited during aging and may modulate neuronal and glial fate determination in the SEZ throughout human life.

## Introduction

The formation of new neurons from stem cells in the subependymal zone (SEZ, also subventricular zone) lining the lateral ventricles persists throughout life in many mammals (Altman, 1969); however, the existence of this neurogenic zone in adult humans is still debated. The adult human SEZ is a four-layered structure, with a monolayer of ependymal cells (layer I), a hypocellular gap (layer II), an astrocytic ribbon of cells (layer III), and a transitory zone (layer IV) (Sanai et al., 2004; Quinones-Hinojosa et al., 2006). The astrocytic ribbon represents a neurogenic niche in which stem cells with astrocyte-like properties reside and can generate transit amplifying precursor cells, which in turn produce neuroblasts that can migrate along the rostral migratory stream into the olfactory bulb (Doetsch et al., 1997). In humans, neuroblasts can also travel along the medial migratory stream and integrate as inhibitory interneurons within the medial prefrontal cortex and frontal lobe, thus putatively contributing to cortical maturation and plasticity (Sanai et al., 2011; Paredes et al., 2016).

Previous reports provide evidence for cell proliferation and the presence of both multipotent precursor cells and immature neurons in the postnatal human SEZ (Weickert et al., 2000; Sanai et al., 2004, 2011; Curtis et al., 2005; Quinones-Hinojosa et al., 2006; Barry et al., 2015). While some groups find little evidence for a rostral migratory stream or significant cellular proliferation in the human SEZ after the early postnatal period (Arellano and Rakic, 2011; Sanai et al., 2011; Dennis et al., 2016), others demonstrate the existence of a rostral migratory stream in humans as well as the presence of proliferating cells and immature neurons in the adult SEZ (Curtis et al., 2007; Kam et al., 2009; Tepavcevic et al., 2011; Wang et al., 2011; Maheu et al., 2015). Furthermore, SEZ precursor cells derived from elderly humans have the capacity to generate neurons and glia *in vitro* (Kukekov et al., 1999; Leonard et al., 2009; van Strien et al., 2014), supporting the continued existence of neural precursor cells even during aging. Thus, there is a clear need for further studies examining the extent of neurogenesis in the human SEZ over the entire adult lifespan.

The proliferation, differentiation and survival of newly formed neurons in the mammalian brain are regulated by various extrinsic factors. Trophic factors, including fibroblast growth factor 2 (FGF2), transforming growth factor alpha (TGFα) and epidermal growth factor (EGF), promote neural cell proliferation *in vivo* and *in vitro* (Tao et al., 1997; Gregg et al., 2001; Doetsch et al., 2002). Lack of TGFα and EGF receptor (EGFR, also ErbB1) results in early postnatal neurodegeneration of the forebrain and reduced precursor cells in the rodent SEZ (Tropepe et al., 1997; Sibilia et al., 1998). In the human SEZ, some essential trophic factors are expressed during postnatal life, with a significant down-regulation observed for EGFR and Erb-B2 receptor tyrosine kinase 4 (ErbB4) mRNAs after infancy (Weickert et al., 2000; Chong et al., 2008; Werry et al., 2010). Further study of key trophic factors and their receptors in the human SEZ throughout adulthood would provide a better understanding of endogenous growth factor signaling as humans grow old and neuroplasticity becomes limited.

In this study, we determined the extent of proliferation, neurogenesis and gliogenesis across adulthood using targeted anatomical dissection of the human SEZ, quantitative reverse transcriptase polymerase chain reaction (qRT-PCR) and immunohistochemistry. We also examined how the expression of EGF- and FGF-related trophic factors changes within this neurogenic zone during aging and ascertained whether these changes correlate with indices of proliferation and cell differentiation. We hypothesized that (1) proliferation and neurogenesis markers will decrease with age in the human SEZ; (2) astrocyte and microglial markers will increase throughout adulthood; (3) expression of trophic factors and their receptors will decrease with age and correlate with reduced levels of proliferation and neurogenesis.

## Materials and Methods

### Human Post-Mortem Brain Samples

Tissue from the anterior caudate of 50 healthy individuals between the ages of 21 and 103 years was obtained from the Stanley Medical Research Institute and New South Wales Brain Tissue Resource Centre. The brain banks have strict exclusion criteria (e.g., infectious diseases such as hepatitis B and C, HIV and Creutzfeldt-Jakob disease) and perform detailed neuropathological examination prior to giving out brain tissue for research purposes. This study was carried out in accordance with the Declaration of Helsinki after review at the University of New South Wales (Sydney, Australia; HREC 12435 and HC 16442). Cases had no known history of psychiatric symptoms or substance abuse and showed no significant neuropathology on post-mortem examination. The cohort consisted of 9 females and 41 males, with an average age of 52 years, average brain pH of 6.59 and average post-mortem interval (PMI) of 29 h. Fresh-frozen tissue containing the rostral caudate was obtained for all cases for mRNA expression analysis, while fixed tissue for immunohistochemistry was available for a subset of the full cohort (Supplementary Table S1).

### Processing of Brain Tissue

Fresh-frozen caudate tissue was sectioned from ∼2 cm coronal blocks on a Leica CM3050 S cryostat, taking 20 × 60 μm sections interspersed with 10 × 14 μm sections. SEZ tissue was dissected from the caudate nucleus while frozen over dry ice from 60 μm thick sections, ∼2 mm deep to the surface of the lateral ventricle. For each case, tissue was dissected from three sets of 3–4 adjacent 60 μm sections spaced ∼1340 μm apart (Supplementary Figure S1) to give 10 sections per case (∼40 mg tissue total). Formalin-fixed caudate tissue was cut into 50 μm floating sections and stored in 10% paraformaldehyde until immunostaining.

### RNA Extraction and cDNA Synthesis

Total RNA was extracted for all cases using Trizol (Life Technologies). RNA quality and concentration were assessed with Agilent Technologies 2100 Bioanalyzer and Nanodrop ND-1000 spectrophotometer. The average RNA integrity number (RIN) was 7. cDNA was synthesized from 3 μg total RNA per case using SuperScript^®^ First-Strand Synthesis kit and random hexamers (Life Technologies).

### Assessment of mRNA Expression of Neurogenesis, Gliogenesis, and Trophic Factor Makers Using Quantitative Reverse Transcription Polymerase Chain Reaction

mRNA levels were measured by TaqMan Gene Expression Assays (Applied Biosystems, Supplementary Table S2) for proliferating cells (Ki67), stem/precursor cells [glial fibrillary acidic protein delta (GFAPδ), achaete-scute homolog 1 (ASCL1, also MASH1)], immature neurons [doublecortin (DCX)], glial cells [pan-glial fibrillary acidic protein (pan-GFAP), vimentin (VIM), oligodendrocyte lineage transcription factor 2 (Olig2), ionized calcium-binding adapter molecule 1 (IBA1, also AIF1)], EGF- and FGF-related factors (EGF, TGFα, and FGF2) and their receptors [EGFR, ErbB4, FGF receptor 1 (FGFR1)] using an ABI Prism 7900HT fast real-time PCR system and a 384-well format. All measurements from each subject were performed in duplicate and relative quantities were determined from a seven-point standard curve of pooled cDNA. qRT-PCR data were captured with sequence detector software (SDS version 2.4, Applied Biosystems). SDS software plotted real-time fluorescence intensity and the threshold was set within the linear phase of the amplification profiles. The no template controls did not produce a signal for any mRNA examined. Expression of two housekeeping genes, TATA-box binding protein and ubiquitin C, was used to calculate the normalizing factor for gene expression (geometric mean), and neither of these mRNAs nor the geometric mean correlated significantly with age (all *p* > 0.05, Supplementary Table S3).

### Localization of Proliferative Cells in the SEZ Using Immunohistochemistry

To determine the presence and distribution of dividing cells in the human SEZ, immunohistochemistry was performed for the proliferation marker Ki67 on 50 μm fixed floating sections using a standard 3,3′-diaminobenzidine protocol as previously described (Weickert et al., 2000; Allen et al., 2015). Briefly, tissue sections were washed in phosphate buffered saline (137 mM NaCl, 2.7 mM KCl, 8 mM Na_2_HPO_4_, 2 mM KH_2_PO_4_, pH 7.4) and placed in 0.75% H_2_O_2_ and 75% methanol for 20 min to block endogenous peroxidase activity at room temperature. Antigen retrieval was performed by heating up to 98°C for 10 min in 10 mM sodium citrate buffer (pH 6.0). After rinsing, sections were incubated with 10% normal goat serum in diluent (0.05% bovine serum albumin, 0.3% Triton X-100 in phosphate buffered saline) for 1 h at room temperature. Primary anti-Ki67 antibody (1:500 in diluent; Abcam, ab15580) was applied overnight at 4°C prior to washing followed by secondary antibody application (1:500 goat anti-rabbit IgG biotinylated, Vector Laboratories), further washing and application of avidin-biotin-peroxidase complex reagent (Vectastain kit, Vector Laboratories) for 1 h at room temperature. Sections were washed and chromagen (0.05% 3,3′-diaminobenzidine, 0.003% H_2_O_2_ in phosphate buffered saline) applied until a reaction was visible. Sections were mounted on glass slides, dehydrated in ethanol, counterstained with thionin, cleared in xylene and cover slipped with Permount. Control slides were incubated in diluent without primary antibody and did not produce any specific signal.

### Quantification of Proliferative Cells in the SEZ

All Ki67 immunoreactive cells in the SEZ were counted from three immunostained sections per individual, except for two individuals for which only two sections were available. The SEZ was defined as the layer between the monolayer of ependymal cells and the transitional zone, including the hypocellular gap and astrocytic ribbon of cells, along the caudate side of the lateral ventricle. Positive cells were identified as those having dark brown nuclear staining. Cells were counted using a Nikon Eclipse 80i light microscope (Coherent Scientific) at 20× magnification initially, and immunopositive cells were confirmed at 40× magnification. The total number of cells counted per slide was normalized to total length of the SEZ (on average 14.4 ± 6.8 mm SD) and averaged across all immunostained sections.

### Statistics

Statistical analyses were performed using IBM SPSS Statistics Version 21 and GraphPad Prism Version 6.0 B. Population outliers were defined as points lying outside of a 95% prediction interval from the linear regression line (0–3 individuals per target gene). Data were tested for normality using the Shapiro–Wilk test. Pearson’s product-moment correlations were used to investigate the relationship of demographic variables (age, brain pH, PMI, and RIN) to each other and target gene expression. Results were considered as significant at an α level of *p* ≤ 0.05. Pearson’s product-moment or semi-partial correlations were used to analyze age-related changes in target gene expression and their relationship to markers of cell proliferation and differentiation. When semi-partial correlations were performed, the semi-partial correlation coefficient sr is reported. The Benjamini-Hochberg method for false discovery rate correction was applied to adjust for multiple comparisons in gene-gene correlation analyses and significance was set to an α level of *p* ≤ 0.018. Sex specific effects on target gene expression were assessed by independent *t*-tests, Mann–Whitney *U* tests or one-way analysis of variance controlling for RIN or PMI as appropriate, and sex did not show a significant effect on mRNA expression for any target examined (data not shown). Mean data are presented for analysis of Ki67 immunohistochemistry. Spearman’s rank correlation was used to determine age-related changes in number of Ki67 immunoreactive cells.

## Results

### Correlations between Demographic Variables and Target Gene Expression

The relationships between age and other demographic variables (brain pH, PMI, and RIN) were assessed in the RNA cohort. Age significantly correlated with brain pH (*r* = -0.43, *p* = 0.002), but age did not correlate with PMI (*r* = -0.05, *p* = 0.69), or RIN (*r* = -0.19, *p* = 0.18). There was no significant relationship between brain pH and RIN (*r* = 0.21, *p* = 0.13) or PMI (*r* = 0.24, *p* = 0.09), thus, brain pH was not considered as a covariate during further analyses. PMI did not correlate with RIN (*r* = 0.03, *p* = 0.80).

Detailed statistical data for the relationship of demographic variables to target gene expression are presented in Supplementary Table S4. Briefly, DCX mRNA positively correlated with brain pH (*r* = 0.41, *p* = 0.003) and RIN (*r* = 0.38, *p* = 0.006). Brain pH negatively correlated with EGFR (*r* = -0.40, *p* = 0.004), FGF2 (*r* = -0.36, *p* = 0.01) and FGFR1 mRNAs (*r* = -0.29, *p* = 0.04), whereas PMI showed a negative relationship to EGFR (*r* = -0.36, *p* = 0.009), FGF2 (*r* = -0.35, *p* = 0.01), and pan-GFAP mRNAs (*r* = -0.29, *p* = 0.04). To account for a possible impact of these demographic variables, PMI or RIN were used as covariates in semi-partial correlations to analyze age-related changes in expression of target genes and their relationship to markers of proliferation and cell differentiation.

### Cell Proliferation and Immature Neuron Markers Declined with Age in the Human SEZ

**FIGURE 1:**
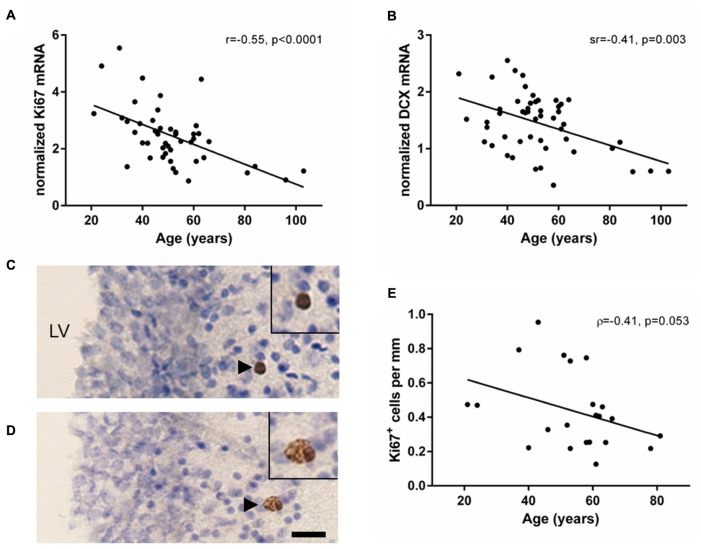
**Markers of cell proliferation and immature neurons decreased with age in the human SEZ.** Cell proliferation (Ki67) and immature neuron (DCX) transcripts were measured by qRT-PCR and normalized to the geometric mean of two housekeeping genes. Ki67 **(A)** and DCX mRNAs **(B)** were negatively correlated with age in the SEZ of individuals ranging from 21 to 103 years of age. Ki67 immunohistochemistry was performed in a subset of individuals of the entire cohort ranging from 21 to 81 years of age. Ki67 immunoreactive cells occurred individually, as doublets or small clusters in the lateral wall of the lateral ventricle **(C,D)**. The total number of Ki67 immunoreactive cells in three immunostained sections were counted and normalized to total length of the SEZ. The mean density of Ki67 immunoreactive cells decreased with age; however, this correlation was just at statistical significance **(E)**. Scale bars = 20 μm. LV, lateral ventricle; r, Pearson’s product-moment correlation coefficient; ρ, Spearman’s rank correlation coefficient; sr, semi-partial correlation coefficient.

Transcript levels of the cell proliferation marker Ki67 were reliably detected in the human SEZ from young to aged adulthood (21–103 years of age). Ki67 mRNA showed a significant negative correlation with age (*r* = -0.55, *p* < 0.0001; **Figure 1A**). DCX mRNA was detected in all samples and also demonstrated a significant negative correlation with age (sr = -0.41, *p* = 0.003; **Figure 1B**); however, DCX and Ki67 mRNAs did not correlate with each other (sr = 0.05, *p* = 0.73).

Ki67 immunoreactivity was qualitatively detected in the nucleus of cells, typically below the ependyma in the hypocellular gap and astrocytic ribbon of cells (**Figures 1C,D**; black arrowheads). An average of 5.5 cells (±4.3 SD) was counted per section, with at least five Ki67 immunoreactive cells per individual. Ki67 immunoreactive cells were found individually (57% of Ki67^+^ cells), in closely associated pairs (34% of Ki67^+^ cells), and occasionally in groups of up to 4 cells (9% of Ki67^+^ cells). The density of Ki67 immunoreactive cells in the human SEZ decreased during aging; however, this was just outside the level of statistical significance (ρ = -0.41, *p* = 0.053; **Figure 1E**).

### Expression of Stem and Precursor Cell Markers Remained Stable in the Aging SEZ

**FIGURE 2:**
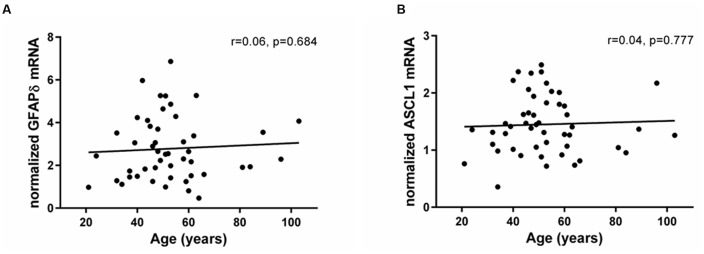
**Expression of stem and precursor cell markers in the SEZ remained stable throughout adulthood.** Stem cell (GFAPδ) and neuronal precursor (ASCL1) transcripts were assessed by qRT-PCR and normalized to the geometric mean of two housekeeping genes. GFAPδ **(A)** and ASCL1 mRNAs **(B)** were reliably detected in all individuals and remained stable during aging. r, Pearson’s product-moment correlation coefficient.

In contrast to the significant changes in Ki67 and DCX expression, mRNA levels encoding the δ-isoform of GFAP, associated with quiescent cells with stem cell-like properties, did not change across the adult lifespan (*r* = 0.06, *p* = 0.68; **Figure 2A**). In support of stable stem cell marker transcripts, we found that the neuronal precursor marker ASCL1 was expressed in all samples and showed no change with age (*r* = 0.04, *p* = 0.77; **Figure 2B**). Ki67 mRNA levels neither correlated with GFAPδ nor with ASCL1 mRNAs (GFAPδ: *r* = 0.15, *p* = 0.30; ASCL1: *r* = 0.20, *p* = 0.17).

### Microglia Expression Increased in the Human SEZ throughout Adulthood

**FIGURE 3:**
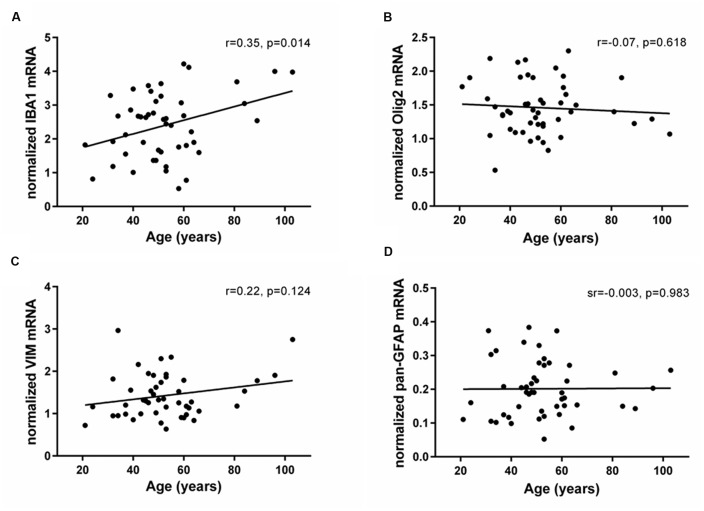
**Expression of glial cell markers in the human SEZ during aging.** Transcript levels of microglia (IBA1), oligodendrocytes (Olig2), immature astrocytes (VIM) and mature astrocytes (pan-GFAP) were assessed by qRT-PCR and normalized to the geometric mean of two housekeeping genes. IBA1 expression **(A)** significantly increased throughout adulthood, while Olig2 **(B)**, VIM **(C),** and pan-GFAP mRNAs **(D)** were unchanged during aging. r, Pearson’s product-moment correlation coefficient; sr, semi-partial correlation coefficient.

The mRNAs for glial lineage markers including IBA1 (microglia), Olig2 (oligodendrocytes), VIM (immature astrocytes) and pan-GFAP (mature astrocytes) were expressed in the human SEZ. IBA1 mRNA positively correlated with age (*r* = 0.35, *p* = 0.014; **Figure 3A**), whereas Olig2, VIM and pan-GFAP demonstrated no age-related change in their transcript levels (all *p* ≥ 0.05, **Figures 3B–D**). There were no relationships between Ki67 mRNA and IBA1, Olig2, VIM, or pan-GFAP transcript levels (all *p* ≥ 0.018, **Table 1**). DCX expression was positively correlated with Olig2 mRNA (sr = 0.37, *p* = 0.008), whereas VIM transcripts showed a negative relationship with DCX expression (sr = -0.41, *p* = 0.003).

**Table 1:** Correlations between expression of glial cell, proliferation, and immature neuron markers in the human SEZ.

	Ki67		DCX (RIN)	
	*r*/sr	*p*-value	sr	*p*-value
pan-GFAP (PMI)	0.295	0.049	-0.282	0.052
IBA1	-0.036	0.815	-0.291	0.045
Olig2	0.190	0.201	**0.377**	**0.008**
VIM	-0.019	0.903	-**0.419**	**0.003**

*Confounding demographic variables (in brackets) were considered as covariates in semi-partial correlation analyses. The significance was adjusted by the Benjamini-Hochberg method for false discovery rate correction and set to an α level of p ≤ 0.018. PMI, post-mortem interval; r, Pearson’s product-moment correlation coefficient; RIN, RNA integrity number; sr, semi-partial correlation coefficient. Bold type = p ≤ 0.018.*

### Expression of EGFR and FGF2 Increased with Age in the Human SEZ

**FIGURE 4:**
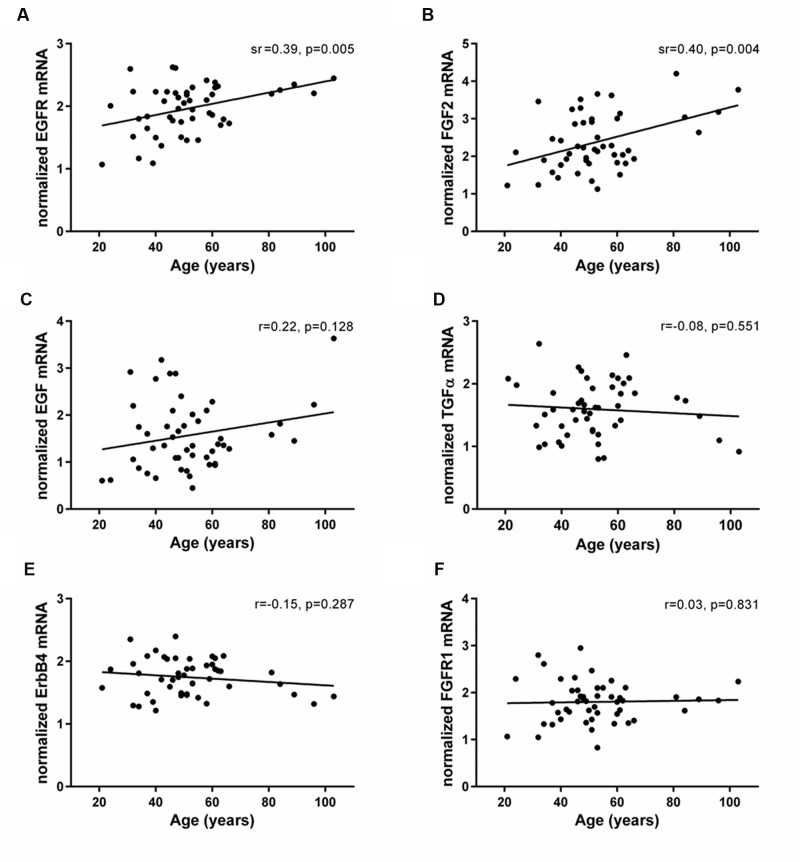
**Expression of EGF- and FGF-related family members in the human SEZ throughout adulthood.** Transcript levels of EGF- and FGF-related growth factors (EGF, TGFα, and FGF2) and receptors (EGFR, ErbB4, and FGFR1) were measured by qRT-PCR and normalized to the geometric mean of two housekeeping genes. EGFR **(A)** and FGF2 mRNAs **(B)** were positively correlated with age in the human SEZ, whereas expression of EGF **(C)**, TGFα **(D)**, ErbB4 **(E),** and FGFR1 **(F)** remained stable throughout adult life. r, Pearson’s product-moment correlation coefficient; sr, semi-partial correlation coefficient.

The mRNAs of EGF- and FGF-related trophic factors (EGF, TGFα, and FGF2) and receptors (EGFR, ErbB4, and FGFR1) were reliably detected in the human SEZ. Transcript levels of EGFR and FGF2 were positively correlated with age (EGFR: sr = 0.39, *p* = 0.005; FGF2: sr = 0.40, *p* = 0.004; **Figures 4A,B**). In contrast, the mRNAs encoding two EGFR ligands, EGF and TGFα, showed no age-related changes in the adult SEZ (EGF: *r* = 0.22, *p* = 0.12; TGFα: *r* = -0.08, *p* = 0.55; **Figures 4C,D**), nor were the mRNA levels of the other two growth factor receptors, ErbB4 and FGFR1, altered with age (ErbB4: *r* = -0.15, *p* = 0.28; FGFR1: *r* = 0.03, *p* = 0.83; **Figures 4E,F**).

### Relationship of EGF- and FGF-Related Transcripts to Expression of Proliferation, Neuronal and Glial Cell Markers in the Human SEZ

**FIGURE 5:**
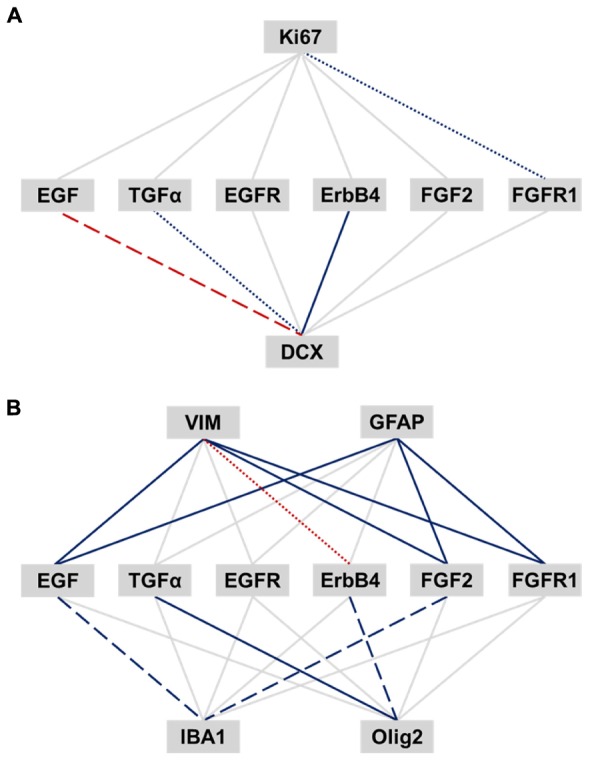
**Relationship of EGF- and FGF-related transcripts to expression of adult neurogenesis and gliogenesis markers in the human SEZ.** Pearson’s product-moment and semi-partial correlations were performed between EGF- and FGF-related transcripts and markers of adult neurogenesis **(A)** and gliogenesis **(B)**. The significance was adjusted by the Benjamini-Hochberg method for false discovery rate correction and set to an α level of *p* ≤ 0.018. Significance levels are displayed by differently dashed lines (*p* > 0.018: gray solid line; *p* < 0.018: colored round dot line; *p* < 0.0036: colored dashed line; *p* < 0.00036: colored solid line). Red lines represent negative correlation, and blue lines positive correlations.

We correlated EGF- and FGF-related transcripts to expression of cell proliferation (Ki67), neuronal (DCX) and glial cell markers (VIM, pan-GFAP, IBA1, and Olig2) within the human SEZ (**Figures 5A,B**). Ki67 mRNA was positively correlated with FGFR1 expression (*r* = 0.36, *p* = 0.012). TGFα and ErbB4 mRNAs positively correlated with DCX transcript levels, whereas EGF mRNA was negatively correlated with DCX expression (TGFα: sr = 0.38, *p* = 0.007; ErbB4: sr = 0.55, *p* < 0.0001; EGF: sr = -0.41, *p* = 0.003).

VIM mRNA was positively correlated with EGF, FGF2, and FGFR1 transcript levels, whereas ErbB4 mRNA showed a negative correlation with VIM expression (EGF: *r* = 0.67, *p* < 0.0001; FGF2: sr = 0.56, *p* < 0.0001; FGFR1: *r* = 0.68, *p* < 0.0001; ErbB4: *r* = -0.41, *p* = 0.005). Pan-GFAP mRNA was positively correlated with EGF, FGF2, and FGFR1 transcript levels (EGF: sr = 0.68, *p* < 0.0001; FGF2: sr = 0.63, *p* < 0.0001; FGFR1: sr = 0.73, *p* < 0.0001). Olig2 transcript levels showed a positive relationship with TGFα and ErbB4 expression (TGFα: *r* = 0.79, *p* < 0.0001; ErbB4: *r* = 0.42, *p* = 0.003), whereas IBA1 mRNA positively correlated with EGF and FGF2 expression (EGF: *r* = 0.47, *p* = 0.001; FGF2: sr = 0.44, *p* = 0.002).

## Discussion

**FIGURE 6:**
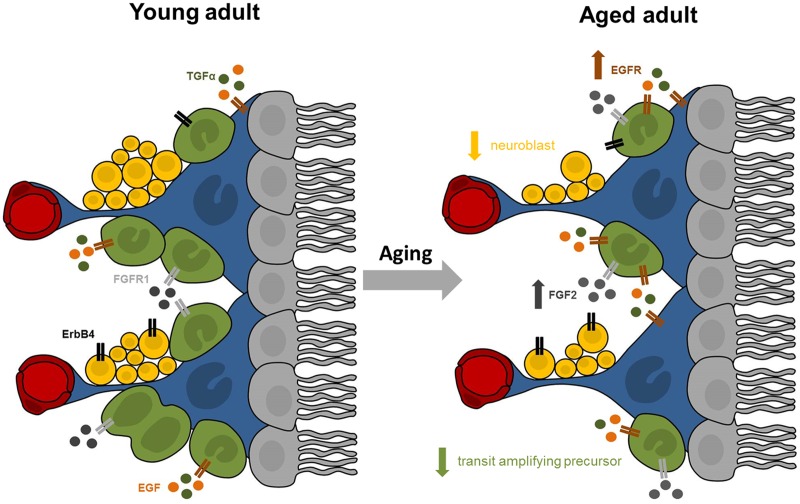
**Summary of proposed age-related changes in the human SEZ.** The adult SEZ is organized below a ciliated ependymal monolayer (in gray) and includes quiescent stem cells with astrocyte-like properties (in blue), transit amplifying precursor cells (in green), neuroblasts (in yellow), and blood vessels (in red). Our results indicate that the aged SEZ has a significantly reduced number of transit amplifying precursors and neuroblasts, while the number of stem cells remained stable. These age-related changes may be modulated by altered expression of growth factors and their receptors. Expression of EGFR (in brown) was significantly increased with advanced age, while transcript levels of FGFR1 (in light gray) and ErbB4 (in black) were unchanged. The major endogenous ligands for EGFR are EGF (in orange) and TGFα (in dark green), whose expression remained stable in the aging SEZ. Expression of FGF2 (in dark gray), primary ligand for FGFR1, significantly increased with age.

While the existence and extent of cell proliferation in the adult human SEZ has been debated, our data provided affirmative evidence of putatively dividing cells in this unique anatomical region throughout adulthood. The expression of Ki67 and DCX mRNAs in the SEZ declined across nine decades of life, while the microglia transcript increased and the expression of stem/precursor, astrocyte and oligodendrocyte cell markers appeared to remain stable. Our study showed for the first time that the mRNA expression of trophic factors and their receptors, rather than being down-regulated with age as hypothesized, were in fact either unchanged or increased in the human SEZ (results are summarized in **Figure 6**). This suggests that decreased endogenous synthesis of trophic factors is not likely to be the reason for the putative diminution in cell genesis and neuronal differentiation during aging. Although correlation analyses only provide clues as to which factors may be active participants, our results indicated that the mRNA encoding FGFR1, but not FGF2 itself, is linked to overall cell proliferation. Our results demonstrated that TGFα and ErbB4 transcript levels may both relate to neuronal and oligodendroglial determination, while EGF, FGF2, and FGFR1 transcript levels were positively associated with mRNAs indicative of immature and fully differentiated astrocytes. EGF and FGF2 expression positively correlated with microglial marker mRNA in the human SEZ, consistent with a putative proliferative or survival impact on this subpopulation.

### Cell Proliferation in the Adult Human SEZ

In order for new neurons to be generated throughout adult life, quiescent stem cells residing in the human SEZ need to re-enter the cell cycle. Ki67 is expressed in the cell cycle during all but the *G*_0_ and early *G*_1_ phases and can be used to provide evidence of a cell engaged in proliferation (Gerdes et al., 1983). A previous report found a 14-fold reduction in the ratio of Ki67 positive cells to total cell number from infancy into adulthood in the human SEZ of the third ventricle (Dahiya et al., 2011). Consistent with this, we found a sevenfold reduction in Ki67 mRNA levels in the post-mortem SEZ of individuals over 90 years as compared to 20-year old young adults. Our report of putatively dividing cells accords with findings of proliferating cell nuclear antigen labeled cells in the human SEZ of individuals from 69 to 87 years of age (Curtis et al., 2005) and indicates that cell proliferation continues into old age rather than virtually ceasing in toddlerhood as previously suggested (Sanai et al., 2011; Wang et al., 2011).

### Neuronal Fate of Newly Born Cells in the Adult SEZ

The astrocytic ribbon of the human SEZ contains quiescent stem cells with astrocyte-like properties (Sanai et al., 2004; van den Berge et al., 2010), and explants from the adult SEZ are capable of generating astrocytes, oligodendrocytes, and neurons *in vitro* (Kukekov et al., 1999; Leonard et al., 2009). In young adult mice, almost all Ki67 immunoreactive cells are positive for precursor markers, nestin and sex determining region Y box 2, as well as markers of the neuronal lineage, DCX and ASCL1 (Bouab et al., 2011). ASCL1 expressing precursors were found to decline by 79% in the SEZ of aged mice compared to young adults (Enwere et al., 2004). We did not find a change in ASCL1 transcripts in the human SEZ, suggesting that neuronal precursors do reside but may not decline throughout adulthood, which is further supported by stable expression of the stem cell marker GFAPδ.

Paredes et al. (2016) could not detect DCX-positive immature neurons after 2 years of age; however, other studies show that DCX-positive immature neurons exist in the adult and aged human SEZ (Tepavcevic et al., 2011; Maheu et al., 2015), which aligns with our qRT-PCR results. Although co-localization of cell proliferation markers with the neuron-specific class III β-tubulin and DCX in the adult macaque and human SEZ suggest that proliferating cells produce immature neurons (Kornack and Rakic, 2001; Wang et al., 2011), we found that proliferative events and neuronal differentiation may not be tightly coupled in the adult human SEZ. Our results indicated that an age-related decrease in the genesis of neural precursors may not be the direct result of a diminished pool of stem cells or possibly even reduced precursor proliferation, suggesting a role for other biological processes that limit neuronal differentiation and survival during aging.

### Expression of Glial Cell Markers in the Human SEZ across the Adult Lifespan

While the majority of dividing cells in the rodent SEZ display characteristics of immature neurons (42% of Ki67^+^ cells are DCX^+^, 87% of 5-bromo-2′deoxyuridine^+^ cells are ASCL1^+^) (Bouab et al., 2011), SEZ precursor proliferation may also generate cells committed to glial lineages. While our findings accord with a previous study indicating that the density of SEZ astrocytes is unchanged in middle-aged to elderly mice (Luo et al., 2006), others demonstrate an age-related increase in GFAP immunostaining and reactive astrocytes in the rodent SEZ (Peretto et al., 2005; Capilla-Gonzalez et al., 2014). Astrocytes promote precursor proliferation and neuronal fate specification (Lim and Alvarez-Buylla, 1999; Song et al., 2002), whereas our results imply that increased astrocyte mRNA expression may block neuronal determination. Aged mice show an increased percentage of 5-bromo-2′deoxyuridine/Olig2 immunoreactive cells, suggesting that the production of oligodendrocytes may be augmented or, as our results support, that the proportion of proliferating cells committed to the oligodendroglial lineage may change across life (Capilla-Gonzalez et al., 2013). While our report of increased IBA1 transcript levels during aging supports several previous studies (DiPatre and Gelman, 1997; Conde and Streit, 2006; Frank et al., 2006; Capilla-Gonzalez et al., 2014), others suggest attenuated microglia generation during postnatal life with fewer microglia produced when cultured from the adult SEZ than when cultured from the neonatal SEZ (Marshall et al., 2008). Although microglia can be harmful or supportive for adult neurogenesis depending on their activation state and functional phenotype (Ekdahl et al., 2009), our results indicated that the age-related increase in IBA1 mRNA expression in the human SEZ occurs alongside decreased cell proliferation and neuronal differentiation and may be uniquely regulated.

### Expression of EGF- and FGF-Related Family Members in the Aging SEZ and Their Putative Regulatory Role in Cell Proliferation and Fate Determination

*In vitro* and *in vivo* studies provide substantial evidence that precursor cells in neurogenic regions require trophic factors for cell proliferation, differentiation, migration, and survival (Craig et al., 1996; Gregg et al., 2001; Doetsch et al., 2002). Previous studies show an age-related developmental decline in EGFR expression in the human SEZ (Weickert et al., 2000; Chong et al., 2008), whereas EGFR transcripts in our cohort significantly increased with advanced age. Other reports show that FGF2 mRNA and protein levels are unchanged in the aging SEZ (Frinchi et al., 2010; Werry et al., 2010), while we detected significantly increased FGF2 mRNA levels in the human SEZ. These temporal differences between EGFR and FGF2 expression levels may be attributed to species differences, differential detection of multiple alternatively spliced variants or age ranges studied (Ilekis et al., 1995; Reiter and Maihle, 1996; Arnaud et al., 1999). Although exogenous stimulation of adult stem cells with FGF2 maintains precursor cells in the cell cycle (Gritti et al., 1996), FGF2-deficient mice show no reduction in precursor proliferation, but demonstrate impairments in neuronal differentiation (Werner et al., 2011). Our results suggest that local FGF2 production may not limit cell proliferation in the human SEZ and indicate that both, FGF2 and FGFR1, may co-operate to promote glial differentiation and maintenance.

ErbB4 expression was previously shown to decline by ∼85% after the first year of birth in the human SEZ (Chong et al., 2008) and we did not detect any further age-related changes in ErbB4 transcripts throughout adulthood. The positive relationship between ErbB4 and DCX mRNA levels aligns with previous findings showing that this receptor is involved in directing neuroblast migration during development (Anton et al., 2004; Ghashghaei et al., 2006).

TGFα mRNA expression is 170 times higher than EGF mRNA levels in the rodent striatum which included the SEZ (Lazar and Blum, 1992). EGF and TGFα mRNAs showed similar expression levels in our human cohort and both remained stable throughout the adult lifespan. While previous rodent studies indicate that TGFα promotes glial differentiation (Weickert and Blum, 1995; Tropepe et al., 1997), our current study in human brains supports a role for TGFα in facilitating neuronal determination. We further speculate that EGF may be the *in situ* factor in the adult human SEZ in blocking neuronal fate in favor of astrocytic differentiation. This role for EGF would be consistent with a rodent study reporting that infusion of EGF decreases the total number of newborn neurons reaching the olfactory bulb and substantially enhances the generation of astrocytes (Kuhn et al., 1997).

## Conclusion

Our study demonstrated a changing pattern of mRNA expression in the SEZ during normal human aging with an overall decrease in proliferation and neuronal maturation markers, but with maintained or increased mRNA expression of several growth factors capable of modulating cell division and differentiation. Understanding how the adult SEZ retains proliferative capacity and the ability to produce putative new neurons is important as stimulation of endogenous neurogenesis or implantation of stem cells have been suggested as therapeutic strategies for brain diseases where neuronal replacement could be beneficial. However, additional studies are required to anatomically map the expression of these factors to cell types and determine the regulatory role of EGF- and FGF-related family members along with other trophic factors involved in adult neurogenesis across the human lifespan.

## Author Contributions

CW contributed to acquisition of data, performing immunohistochemistry, microscopy and qRT-PCRs, statistical analysis and interpretation of data and drafting of the manuscript. SF contributed to the study design including choice of targets, performed RNA extraction, qRT-PCR and microscopy, helped with analysis and interpretation of data and editing of the manuscript. MW dissected the human SEZ, performed RNA extractions and qRT-PCRs and edited the manuscript. GB, KD, and GH contributed to interpretation of data and editing of the manuscript. MJW helped to guide the study design, performed anatomical dissection of the human caudate and edited the manuscript. CSW conceived of the study and contributed to the study design, coordination of the study, analysis and interpretation of data and editing the manuscript. All authors have read and approved the final manuscript.

## Conflict of Interest Statement

The authors declare that the research was conducted in the absence of any commercial or financial relationships that could be construed as a potential conflict of interest.

## Abbreviations

ASCL1: achaete-scute homolog 1 (also MASH1)
DCX: doublecortin
EGF: epidermal growth factor
EGFR: epidermal growth factor receptor (also ErbB1)
ErbB4: Erb-B2 receptor tyrosine kinase 4
FGF2: fibroblast growth factor 2
FGFR1: fibroblast growth factor receptor 1
GFAP: glial fibrillary acidic protein
IBA1: ionized calcium-binding adapter molecule 1 (also AIF1)
Olig2: oligodendrocyte lineage transcription factor 2
PMI: post-mortem interval
qRT-PCR: quantitative reverse transcriptase polymerase chain reaction
RIN: RNA integrity number
SEZ: subependymal zone
TGFα: transforming growth factor alpha
VIM: vimentin.

## References

[B1] Allen K. M. Fung S. J. Shannon Weickert C. (2015). Cell proliferation is reduced in the hippocampus in schizophrenia. *Aust. N. Z. J. Psychiatry* 50 473–480. 10.1177/0004867415589793 26113745 PMC4843086

[B2] Altman J. (1969). Autoradiographic and histological studies of postnatal neurogenesis, IV: cell proliferation and migration in the anterior forebrain, with special reference to persisting neurogenesis in the olfactory bulb. *J. Comp. Neurol.* 137 433–458. 10.1002/cne.901370404 5361244

[B3] Anton E. S. Ghashghaei H. T. Weber J. L. Mccann C. Fischer T. M. Cheung I. D. (2004). Receptor tyrosine kinase ErbB4 modulates neuroblast migration and placement in the adult forebrain. *Nat. Neurosci.* 7 1319–1328. 10.1038/nn1345 15543145

[B4] Arellano J. I. Rakic P. (2011). Neuroscience: gone with the wean. *Nature* 478 333–334. 10.1038/478333a 22012389

[B5] Arnaud E. Touriol C. Boutonnet C. Gensac M. C. Vagner S. Prats H. (1999). A new 34-kilodalton isoform of human fibroblast growth factor 2 is cap dependently synthesized by using a non-AUG start codon and behaves as a survival factor. *Mol. Cell. Biol.* 19 505–514. 10.1128/MCB.19.1.505 9858574 PMC83908

[B6] Barry G. Guennewig B. Fung S. Kaczorowski D. Weickert C. S. (2015). Long non-coding RNA expression during aging in the human subependymal zone. *Front. Neurol.* 6:45 10.3389/fneur.2015.00045 PMC4353253 25806019

[B7] Bouab M. Paliouras G. N. Aumont A. Forest-Berard K. Fernandes K. J. (2011). Aging of the subventricular zone neural stem cell niche: evidence for quiescence-associated changes between early and mid-adulthood. *Neuroscience* 173 135–149. 10.1016/j.neuroscience.2010.11.032 21094223

[B8] Capilla-Gonzalez V. Cebrian-Silla A. Guerrero-Cazares H. Garcia-Verdugo J. M. Quinones-Hinojosa A. (2013). The generation of oligodendroglial cells is preserved in the rostral migratory stream during aging. *Front. Cell. Neurosci.* 7:147 10.3389/fncel.2013.00147 PMC3775451 24062640

[B9] Capilla-Gonzalez V. Cebrian-Silla A. Guerrero-Cazares H. Garcia-Verdugo J. M. Quinones-Hinojosa A. (2014). Age-related changes in astrocytic and ependymal cells of the subventricular zone. *Glia* 62 790–803. 10.1002/glia.22642 24677590 PMC4322944

[B10] Chong V. Z. Webster M. J. Rothmond D. A. Weickert C. S. (2008). Specific developmental reductions in subventricular zone ErbB1 and ErbB4 mRNA in the human brain. *Int. J. Dev. Neurosci.* 26 791–803. 10.1016/j.ijdevneu.2008.06.004 18662768

[B11] Conde J. R. Streit W. J. (2006). Microglia in the aging brain. *J. Neuropathol. Exp. Neurol.* 65 199–203. 10.1097/01.jnen.0000202887.22082.63 16651881

[B12] Craig C. G. Tropepe V. Morshead C. M. Reynolds B. A. Weiss S. Van Der Kooy D. (1996). In vivo growth factor expansion of endogenous subependymal neural precursor cell populations in the adult mouse brain. *J. Neurosci.* 16 2649–2658. 8786441 10.1523/JNEUROSCI.16-08-02649.1996 PMC6578757

[B13] Curtis M. A. Kam M. Nannmark U. Anderson M. F. Axell M. Z. Wikkelso C. (2007). Human neuroblasts migrate to the olfactory bulb via a lateral ventricular extension. *Science* 315 1243–1249. 10.1126/science.1136281 17303719

[B14] Curtis M. A. Penney E. B. Pearson J. Dragunow M. Connor B. Faull R. L. (2005). The distribution of progenitor cells in the subependymal layer of the lateral ventricle in the normal and Huntington’s disease human brain. *Neuroscience* 132 777–788. 10.1016/j.neuroscience.2004.12.051 15837138

[B15] Dahiya S. Lee D. Y. Gutmann D. H. (2011). Comparative characterization of the human and mouse third ventricle germinal zones. *J. Neuropathol. Exp. Neurol.* 70 622–633. 10.1097/NEN.0b013e31822200aa 21666496 PMC3127083

[B16] Dennis C. V. Suh L. S. Rodriguez M. L. Kril J. J. Sutherland G. T. (2016). Human adult neurogenesis across the ages: an immunohistochemical study. *Neuropathol. Appl. Neurobiol.* 10.1111/nan.12337 [Epub ahead of print]. PMC5125837 27424496

[B17] DiPatre P. L. Gelman B. B. (1997). Microglial cell activation in aging and Alzheimer disease: partial linkage with neurofibrillary tangle burden in the hippocampus. *J. Neuropathol. Exp. Neurol.* 56 143–149. 10.1097/00005072-199702000-00004 9034367

[B18] Doetsch F. Garcia-Verdugo J. M. Alvarez-Buylla A. (1997). Cellular composition and three-dimensional structure of the subventricular germinal zone in the adult mammalian brain. *J. Neurosci.* 17 5046–5061. 9185542 10.1523/JNEUROSCI.17-13-05046.1997 PMC6573289

[B19] Doetsch F. Petreanu L. Caille I. Garcia-Verdugo J. M. Alvarez-Buylla A. (2002). EGF converts transit-amplifying neurogenic precursors in the adult brain into multipotent stem cells. *Neuron* 36 1021–1034. 10.1016/S0896-6273(02)01133-9 12495619

[B20] Ekdahl C. T. Kokaia Z. Lindvall O. (2009). Brain inflammation and adult neurogenesis: the dual role of microglia. *Neuroscience* 158 1021–1029. 10.1016/j.neuroscience.2008.06.052 18662748

[B21] Enwere E. Shingo T. Gregg C. Fujikawa H. Ohta S. Weiss S. (2004). Aging results in reduced epidermal growth factor receptor signaling, diminished olfactory neurogenesis, and deficits in fine olfactory discrimination. *J. Neurosci.* 24 8354–8365. 10.1523/JNEUROSCI.2751-04.2004 15385618 PMC6729689

[B22] Frank M. G. Barrientos R. M. Biedenkapp J. C. Rudy J. W. Watkins L. R. Maier S. F. (2006). mRNA up-regulation of MHC II and pivotal pro-inflammatory genes in normal brain aging. *Neurobiol. Aging* 27 717–722. 10.1016/j.neurobiolaging.2005.03.013 15890435

[B23] Frinchi M. Di Liberto V. Olivieri M. Fuxe K. Belluardo N. Mudo G. (2010). FGF-2/FGFR1 neurotrophic system expression level and its basal activation do not account for the age-dependent decline of precursor cell proliferation in the subventricular zone of rat brain. *Brain Res.* 1358 39–45. 10.1016/j.brainres.2010.08.083 20816673

[B24] Gerdes J. Schwab U. Lemke H. Stein H. (1983). Production of a mouse monoclonal antibody reactive with a human nuclear antigen associated with cell proliferation. *Int. J. Cancer* 31 13–20. 10.1002/ijc.2910310104 6339421

[B25] Ghashghaei H. T. Weber J. Pevny L. Schmid R. Schwab M. H. Lloyd K. C. (2006). The role of neuregulin-ErbB4 interactions on the proliferation and organization of cells in the subventricular zone. *Proc. Natl. Acad. Sci. U.S.A.* 103 1930–1935. 10.1073/pnas.0510410103 16446434 PMC1413654

[B26] Gregg C. T. Shingo T. Weiss S. (2001). Neural stem cells of the mammalian forebrain. *Symp. Soc. Exp. Biol.* 53 1–19. 12063843

[B27] Gritti A. Parati E. A. Cova L. Frolichsthal P. Galli R. Wanke E. (1996). Multipotential stem cells from the adult mouse brain proliferate and self-renew in response to basic fibroblast growth factor. *J. Neurosci.* 16 1091–1100. 8558238 10.1523/JNEUROSCI.16-03-01091.1996 PMC6578802

[B28] Ilekis J. V. Stark B. C. Scoccia B. (1995). Possible role of variant RNA transcripts in the regulation of epidermal growth factor receptor expression in human placenta. *Mol. Reprod. Dev.* 41 149–156. 10.1002/mrd.1080410205 7654368

[B29] Kam M. Curtis M. A. Mcglashan S. R. Connor B. Nannmark U. Faull R. L. (2009). The cellular composition and morphological organization of the rostral migratory stream in the adult human brain. *J. Chem. Neuroanat.* 37 196–205. 10.1016/j.jchemneu.2008.12.009 19159677

[B30] Kornack D. R. Rakic P. (2001). The generation, migration, and differentiation of olfactory neurons in the adult primate brain. *Proc. Natl. Acad. Sci. U.S.A.* 98 4752–4757. 10.1073/pnas.081074998 11296302 PMC31906

[B31] Kuhn H. G. Winkler J. Kempermann G. Thal L. J. Gage F. H. (1997). Epidermal growth factor and fibroblast growth factor-2 have different effects on neural progenitors in the adult rat brain. *J. Neurosci.* 17 5820–5829. 9221780 10.1523/JNEUROSCI.17-15-05820.1997 PMC6573198

[B32] Kukekov V. G. Laywell E. D. Suslov O. Davies K. Scheﬄer B. Thomas L. B. (1999). Multipotent stem/progenitor cells with similar properties arise from two neurogenic regions of adult human brain. *Exp. Neurol.* 156 333–344. 10.1006/exnr.1999.7028 10328940

[B33] Lazar L. M. Blum M. (1992). Regional distribution and developmental expression of epidermal growth factor and transforming growth factor-alpha mRNA in mouse brain by a quantitative nuclease protection assay. *J. Neurosci.* 12 1688–1697. 1578263 10.1523/JNEUROSCI.12-05-01688.1992 PMC6575894

[B34] Leonard B. W. Mastroeni D. Grover A. Liu Q. Yang K. Gao M. (2009). Subventricular zone neural progenitors from rapid brain autopsies of elderly subjects with and without neurodegenerative disease. *J. Comp. Neurol.* 515 269–294. 10.1002/cne.22040 19425077 PMC2757160

[B35] Lim D. A. Alvarez-Buylla A. (1999). Interaction between astrocytes and adult subventricular zone precursors stimulates neurogenesis. *Proc. Natl. Acad. Sci. U.S.A.* 96 7526–7531. 10.1073/pnas.96.13.7526 10377448 PMC22119

[B36] Luo J. Daniels S. B. Lennington J. B. Notti R. Q. Conover J. C. (2006). The aging neurogenic subventricular zone. *Aging Cell* 5 139–152. 10.1111/j.1474-9726.2006.00197.x 16626393

[B37] Maheu M. E. Devorak J. Freibauer A. Davoli M. A. Turecki G. Mechawar N. (2015). Increased doublecortin (DCX) expression and incidence of DCX-immunoreactive multipolar cells in the subventricular zone-olfactory bulb system of suicides. *Front. Neuroanat.* 9:74 10.3389/fnana.2015.00074 PMC4450175 26082689

[B38] Marshall G. P. Demir M. Steindler D. A. Laywell E. D. (2008). Subventricular zone microglia possess a unique capacity for massive in vitro expansion. *Glia* 56 1799–1808. 10.1002/glia.20730 18661554 PMC4424978

[B39] Paredes M. F. James D. Gil-Perotin S. Kim H. Cotter J. A. Ng C. (2016). Extensive migration of young neurons into the infant human frontal lobe. *Science* 354:aa f70731–7. 10.1126/science.aaf7073 PMC5436574 27846470

[B40] Peretto P. Giachino C. Aimar P. Fasolo A. Bonfanti L. (2005). Chain formation and glial tube assembly in the shift from neonatal to adult subventricular zone of the rodent forebrain. *J. Comp. Neurol.* 487 407–427. 10.1002/cne.20576 15906315

[B41] Quinones-Hinojosa A. Sanai N. Soriano-Navarro M. Gonzalez-Perez O. Mirzadeh Z. Gil-Perotin S. (2006). Cellular composition and cytoarchitecture of the adult human subventricular zone: a niche of neural stem cells. *J. Comp. Neurol.* 494 415–434. 10.1002/cne.20798 16320258

[B42] Reiter J. L. Maihle N. J. (1996). A 1.8 kb alternative transcript from the human epidermal growth factor receptor gene encodes a truncated form of the receptor. *Nucleic Acids Res.* 24 4050–4056. 10.1093/nar/24.20.4050 8918811 PMC146204

[B43] Sanai N. Nguyen T. Ihrie R. A. Mirzadeh Z. Tsai H. H. Wong M. (2011). Corridors of migrating neurons in the human brain and their decline during infancy. *Nature* 478 382–386. 10.1038/nature10487 21964341 PMC3197903

[B44] Sanai N. Tramontin A. D. Quinones-Hinojosa A. Barbaro N. M. Gupta N. Kunwar S. (2004). Unique astrocyte ribbon in adult human brain contains neural stem cells but lacks chain migration. *Nature* 427 740–744. 10.1038/nature02301 14973487

[B45] Sibilia M. Steinbach J. P. Stingl L. Aguzzi A. Wagner E. F. (1998). A strain-independent postnatal neurodegeneration in mice lacking the EGF receptor. *EMBO J.* 17 719–731. 10.1093/emboj/17.3.719 9450997 PMC1170421

[B46] Song H. Stevens C. F. Gage F. H. (2002). Astroglia induce neurogenesis from adult neural stem cells. *Nature* 417 39–44. 10.1038/417039a 11986659

[B47] Tao Y. Black I. B. Dicicco-Bloom E. (1997). In vivo neurogenesis is inhibited by neutralizing antibodies to basic fibroblast growth factor. *J. Neurobiol.* 33 289–296. 10.1002/(SICI)1097-4695(199709)33:3<289::AID-NEU7>3.3.CO;2-X 9298766

[B48] Tepavcevic V. Lazarini F. Alfaro-Cervello C. Kerninon C. Yoshikawa K. Garcia-Verdugo J. M. (2011). Inflammation-induced subventricular zone dysfunction leads to olfactory deficits in a targeted mouse model of multiple sclerosis. *J. Clin. Invest.* 121 4722–4734. 10.1172/JCI59145 22056384 PMC3226002

[B49] Tropepe V. Craig C. G. Morshead C. M. Van Der Kooy D. (1997). Transforming growth factor-alpha null and senescent mice show decreased neural progenitor cell proliferation in the forebrain subependyma. *J. Neurosci.* 17 7850–7859. 9315905 10.1523/JNEUROSCI.17-20-07850.1997 PMC6793925

[B50] van den Berge S. A. Middeldorp J. Zhang C. E. Curtis M. A. Leonard B. W. Mastroeni D. (2010). Longterm quiescent cells in the aged human subventricular neurogenic system specifically express GFAP-delta. *Aging Cell* 9 313–326. 10.1111/j.1474-9726.2010.00556.x 20121722

[B51] van Strien M. E. Sluijs J. A. Reynolds B. A. Steindler D. A. Aronica E. Hol E. M. (2014). Isolation of neural progenitor cells from the human adult subventricular zone based on expression of the cell surface marker CD271. *Stem Cells Transl. Med.* 3 470–480. 10.5966/sctm.2013-0038 24604282 PMC3973708

[B52] Wang C. Liu F. Liu Y. Y. Zhao C. H. You Y. Wang L. (2011). Identification and characterization of neuroblasts in the subventricular zone and rostral migratory stream of the adult human brain. *Cell. Res.* 21 1534–1550. 10.1038/cr.2011.83 21577236 PMC3365638

[B53] Weickert C. S. Blum M. (1995). Striatal TGF-a: Postnatal developmental expression and evidence for a role in the proliferation of subependymal cells. *Dev. Brain Res.* 86 203–216. 10.1016/0165-3806(95)00026-A 7656413

[B54] Weickert C. S. Webster M. J. Colvin S. M. Herman M. M. Hyde T. M. Weinberger D. R. (2000). Localization of epidermal growth factor receptors and putative neuroblasts in human subependymal zone. *J. Comp. Neurol.* 423 359–372. 10.1002/1096-9861(20000731)423:3<359::AID-CNE1>3.0.CO;2-0 10870078

[B55] Werner S. Unsicker K. Von Bohlen Und Halbach O. (2011). Fibroblast growth factor-2 deficiency causes defects in adult hippocampal neurogenesis, which are not rescued by exogenous fibroblast growth factor-2. *J. Neurosci. Res.* 89 1605–1617. 10.1002/jnr.22680 21800348

[B56] Werry E. L. Enjeti S. Halliday G. M. Sachdev P. S. Double K. L. (2010). Effect of age on proliferation-regulating factors in human adult neurogenic regions. *J. Neurochem.* 115 956–964. 10.1111/j.1471-4159.2010.06992.x 20831616

