# Supplementary material for: Decline in Proliferation and Immature Neuron Markers in the Human Subependymal Zone during Aging: Relationship to EGF- and FGF-Related Transcripts
Source: Front Aging Neurosci. 2016 Nov 25;8:274. doi: 10.3389/fnagi.2016.00274 (PMC5123444; doi:10.3389/fnagi.2016.00274)
Supplement: Supplementary file 5 [file Image_1.PDF]

Supplementary Figure 1: Dissection of the SEZ from fresh-frozen human post-mortem brain.

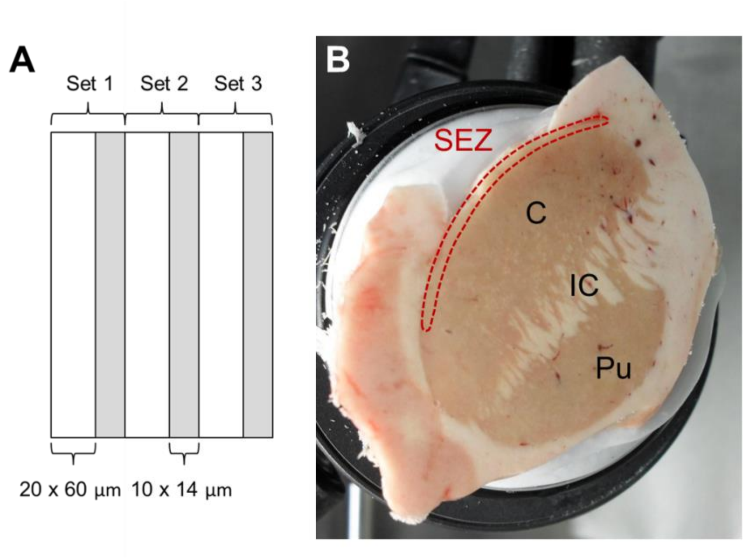

Fresh-frozen tissue was sectioned on a Leica CM3050 S cryostat, taking 20 x 60 µm sections interspersed with 10 x 14 µm sections **(A)**. For each individual, the SEZ, defined by a 2 mm border medial to the caudate, was dissected from 3 sets of 3-4 adjacent 60 µm sections spaced ~1340 µm to give ~40 mg tissue total for RNA extraction **(B)**. C, caudate; IC, internal capsule; Pu, putamen; SEZ, subependymal zone.
